# Changes in liver stiffness measurement using acoustic radiation force impulse elastography after antiviral therapy in patients with chronic hepatitis C

**DOI:** 10.1371/journal.pone.0190455

**Published:** 2018-01-02

**Authors:** Sheng-Hung Chen, Hsueh-Chou Lai, I-Ping Chiang, Wen-Pang Su, Chia-Hsin Lin, Jung-Ta Kao, Po-Heng Chuang, Wei-Fan Hsu, Hung-Wei Wang, Hung-Yao Chen, Guan-Tarn Huang, Cheng-Yuan Peng

**Affiliations:** 1 Graduate Institute of Clinical Medical Science, School of Medicine, China Medical University, Taichung, Taiwan; 2 School of Medicine, China Medical University, Taichung, Taiwan; 3 Division of Hepatogastroenterology, Department of Internal Medicine, China Medical University Hospital, Taichung, Taiwan; 4 College of Chinese Medicine, China Medical University, Taichung, Taiwan; 5 Department of Pathology, China Medical University Hospital, Taichung, Taiwan; National Taiwan University Hospital, TAIWAN

## Abstract

**Background:**

To compare on-treatment and off-treatment parameters acquired using acoustic radiation force impulse elastography, the Fibrosis-4 (FIB-4) index, and aspartate aminotransferase-to-platelet ratio index (APRI) in patients with chronic hepatitis C (CHC).

**Methods:**

Patients received therapies based on pegylated interferon or direct-acting antiviral agents. The changes in paired patient parameters, including liver stiffness (LS) values, the FIB-4 index, and APRI, from baseline to sustained virologic response (SVR) visit (24 weeks after the end of treatment) were compared. Multiple regression models were used to identify significant factors that explained the correlations with LS, FIB-4, and APRI values and SVR.

**Results:**

A total of 256 patients were included, of which 219 (85.5%) achieved SVR. The paired LS values declined significantly from baseline to SVR visit in all groups and subgroups except the nonresponder subgroup (n = 10). Body mass index (*P* = 0.0062) and baseline LS (*P* < 0.0001) were identified as independent factors that explained the LS declines. Likewise, the baseline FIB-4 (*P* < 0.0001) and APRI (*P* < 0.0001) values independently explained the declines in the FIB-4 index and APRI, respectively. Moreover, interleukin-28B polymorphisms, baseline LS, and rapid virologic response were identified as independent correlates with SVR.

**Conclusions:**

Paired LS measurements in patients treated for CHC exhibited significant declines comparable to those in FIB-4 and APRI values. These declines may have correlated with the resolution of necroinflammation. Baseline LS values predicted SVR.

## Introduction

Chronic hepatitis C (CHC) is a major global healthcare challenge [1]. Maintaining a sustained virologic response (SVR) after treatment is the most crucial goal of treatment for CHC. In most resource-limited areas worldwide and most Asian countries, pegylated interferon (pegIFN) and ribavirin (RBV) combination therapy has been the first-line standard of care for CHC for many years.

SVR is approximately equivalent to the cessation of viral replication, necroinflammation, and fibrosis progression. Patients with steady and prolonged virus-clearance states after SVR subsequently develop fewer adverse endpoints such as cirrhosis, decompensation, and hepatocellular carcinoma (HCC) than do those without SVR [2]. However, SVR does not guarantee the prevention of such adverse liver-related endpoints. Therefore, post-SVR care requires programmed surveillance [3]. Before predicting post-SVR endpoints, parameters over the treatment course prior to the occurrence of SVR should be investigated to obtain more insights into CHC.

Most patients who receive a diagnosis of CHC, even those without SVR, benefit from antiviral treatments in terms of the reversal of necroinflammation and fibrosis during and after the treatment course [2**–**4]. In the era of noninvasive liver fibrosis evaluations, patients have exhibited declines in noninvasive indices to a certain extent before the occurrence of SVR [5**–**10]. However, the results of a comparison between SVR and no SVR varied among study cohorts [11]. Because direct-acting antiviral (DAA) therapies can result in high SVR rates, studies on patients receiving pegIFN-based treatments have reported notable observations in non-SVR groups.

In addition to virologic responses, liver fibrosis stages have a considerable effect on CHC. Baseline fibrosis stages are significantly correlated with the development of adverse effects after treatment and risk of adverse liver-related endpoints, but inversely correlated with treatment responses [12].

Among these noninvasive liver fibrosis evaluation tools or indices, elastography-based liver stiffness (LS) measurements (LSMs) in particular are affected early by hepatic necroinflammatory activities [8, 13**–**16], which typically decline concomitantly throughout the treatment course. Moreover, the indices based on the combination of the platelet count and liver enzyme levels are affected by factors predisposed to the kinetics of the platelet count during on- and off-treatment periods [9, 10]. However, because of a diverse range of explaining factors, these evaluation tools or indices have rarely been directly compared before the occurrence of SVR.

This study compared chronological on-treatment and off-treatment patient parameters acquired using acoustic radiation force impulse (ARFI) elastography, the Fibrosis-4 (FIB-4) index, and the aspartate aminotransferase (AST)-to-platelet ratio index (APRI) to evaluate the factors that cause a decline in LS, FIB-4, and APRI values and to estimate the effects of these baseline fibrosis-relevant surrogate parameters on SVR.

## Methods

### Ethics statement

Written informed consent was obtained from all participants. The study protocol was approved by the Research Ethics Committee of China Medical University Hospital (DMR99-IRB-240, DMR101-IRB2-301, CMUH103-REC1-102, and CMUH103-REC1-150) and developed in accordance with the Declaration of Helsinki, 1975.

### Patients

Consecutive patients who had received a diagnosis of CHC at China Medical University Hospital from January 2010 to January 2017 were screened. Patients who received pegIFN-based therapy were enrolled in a prospective cohort study for the diagnostic performance of ARFI elastography in the staging of fibrosis in CHC. Patients who received DAA-based therapy were derived from a cohort of subjects enrolled in DAA clinical trials (registration numbers: NCT02021643, NCT02021656, NCT02105467, NCT02105688, NCT02105701, NCT02170727, NCT02251990, NCT02517515, NCT02517528, and NCT02604017). They underwent liver biopsy owing to the study requirement. CHC infection was determined by positive results for hepatitis C virus (HCV) antibodies (Abbott Laboratories, Abbott Park, IL, USA) for more than 6 months with detectable serum HCV RNA (detection limit: 15 IU/mL; COBAS Ampliprep/COBAS TaqMan HCV test, Roche Diagnostics, Branchburg, NJ, USA). Patient exclusion criteria comprised age < 20 years, decompensated cirrhosis (Child–Turcott–Pugh score of ≥ 7), HCC, primary biliary cirrhosis, primary sclerosing cholangitis, Wilson disease, autoimmune hepatitis, hemochromatosis, extrahepatic cholestasis, alcohol dependence (scored as a total of 2 or more on the CAGE Questionnaire) [17], myeloproliferative disorder, thalassemia, cardiac congestion, blood product transfusion in the preceding 30 days, pregnancy, and a serum creatinine level of > 2.5 mg/dL.

### Blood tests

Complete blood count (Sysmex, Hyogo, Japan) and blood biochemistry (Beckman Coulter, Brea, CA, USA) analyses were performed in the central laboratory of the hospital. The FIB-4 index was calculated using the following formula: age (y) × AST (IU/L) / (platelet count [×10^9^/L] × alanine aminotransferase [ALT; IU/L]^1/2^). The APRI was calculated using the following formula: (AST [IU/L] / upper limit of normal [IU/L]) × 100 / platelet count (10^9^/L) [9, 10].

### LSM using ARFI

Each patient underwent percutaneous right-lobe liver biopsy within 1 hour of blood sampling and baseline LSM after 3 hours of fasting at the treatment baseline. The other LSM was performed at the SVR visit or 24 weeks after the end of treatment (EOT).

ARFI elastography was integrated into an ultrasound system (Acuson S2000 with a Siemens 4C1 curved array, 2.67 MHz for push pulses, and 3.08 MHz for detection pulses; Siemens Medical Solutions, Mountain View, CA, USA). LSMs were obtained using detection pulses and presented as shear wave velocity in meters per second (m/s).

A single hepatologist experienced in using digestive system ultrasonography and blinded to the participants’ data obtained the LSMs. Measurement results were deemed reliable when the interquartile range (IQR) was lower than 30% of the median of 10 successful LSMs and the successful LSM rate was higher than 60%. All other results were deemed unreliable and excluded [14, 15, 18].

### METAVIR scoring

Senior hepatologists performed percutaneous right-lobe liver biopsies. Liver tissue sections were stained using Masson’s trichrome staining, hematoxylin and eosin, and reticulin, and were interpreted by a single experienced pathologist blinded to LSM results and participants’ data. In the histology examination, liver fibrosis (F) was staged as F0 (no fibrosis), F1 (portal fibrosis without septa), F2 (portal fibrosis with a few septa), F3 (numerous septa without cirrhosis), and F4 (cirrhosis). Necroinflammatory activity (A) was graded as A0 (no activity), A1 (mild), A2 (moderate), and A3 (severe). Steatosis was graded as S0 (no fatty metamorphosis), S1 (mild), S2 (moderate), and S3 (severe).

### Antiviral therapy

Patients received pegIFN- or DAA-based therapy. Those who received pegIFN-based therapy were prescribed pegIFN α-2a (Pegasys, Hoffmann-La Roche, Basel, Switzerland) at a dosage of 180 μg/week or pegIFN α-2b (Peg-Intron, Merck & Co., Inc., Kenilworth, NJ, USA) at a dosage of 1.5 μg/kg/week subcutaneously, and were administered oral RBV at a daily dosage of 1000 mg (body weight < 75 kg) or 1200 mg (body weight ≥ 75 kg) for 24 or 48 weeks depending on each patient’s virologic response at week 4 [12]. Doses of RBV (mg/kg/day) during treatment were calculated for each patient.

### Treatment monitoring

Blood sampling was performed as scheduled. For each patient, in addition to HCV RNA load, blood biochemistry and complete blood count were monitored and quantified in the central laboratory of the hospital at weeks 4, 12, 24, 48, and 24 weeks after treatment (SVR visit).

### Clinical endpoints

SVR was defined as undetectable HCV RNA at the EOT and at 24 weeks after the EOT for both the pegIFN- and DAA-based groups. Non-SVR was defined as any detectable HCV RNA at or after the EOT. Rapid virologic response (RVR) was defined as undetectable HCV RNA at week 4 of treatment. The rates of RVR were also calculated.

### Statistical analyses

Data are presented in this paper as the median (IQR) or number (percentage). Between-group and overall differences were estimated using the Mann–Whitney U test and Kruskal–Wallis test for continuous variables and chi-square test or Fisher’s exact test for proportions.

Changes in paired patient parameters (e.g., LS, FIB-4, and APRI values) from baseline to SVR visit were compared using the Wilcoxon signed-rank test. Continuous data are expressed in this paper as medians with IQRs in parentheses. Stepwise multiple linear regression models were used to identify the independent factors that explained the changes in LS, FIB-4, and APRI values.

Variables including age; sex; body mass index (BMI); gamma-glutamyl transpeptidase (γ-GT) level; METAVIR F stages and A grades; platelet count; international normalized ratio (INR) of prothrombin time (PT), hemoglobin level; serum AST, ALT, bilirubin, and creatinine levels; and interleukin-28B polymorphisms were designated as covariates in the regression analyses.

Multiple stepwise binary logistic regressions were used to identify significant and independent factors that correlated with SVR. Receiver operating characteristic (ROC) curve analysis was employed to evaluate and compare the diagnostic performance by using the areas under the ROC curves (AUCs) to dichotomize the SVR and non-SVR groups.

Data were analyzed using SAS version 9.3 (SAS Institute, Inc., Cary, NC, USA) and SPSS version 17.0 for Microsoft Windows (SPSS, Inc., Chicago, IL, USA). A 2-sided *P* value of <0.05 indicated statistical significance.

## Results

### Participant characteristics

A total of 283 patients who had received diagnoses of CHC were screened. Of the 283 patients, 27 were excluded for the following reasons: 5 had unreliable LSMs; 4 and 3 had received diagnoses of alcohol dependence and HCC, respectively; 4 had missing data; and 11 had their treatment discontinued before the EOT.

In the final analysis, 256 patients were included (Table 1). Of these, 121 (47.3%) were men with a median age of 54 years (IQR = 15); 163 (63.7%) were infected with HCV genotypes 1, 4, 5, or 6 and 93 (36.3%) with genotypes 2 or 3; 108 (42.2%) and 103 (40.2%) received pegIFN with RBV for 24 and 48 weeks, respectively; and 45 (17.6%) received DAA-based therapy, of which only 9 (20%) also received RBV.

**Table 1:** Comparisons of patient characteristics between groups with baseline liver stiffness of ≥1.82 and <1.82 m/s.

	Total (n = 256)	LS≥1.82 (n = 85)	LS<1.82 (n = 171)	*P* value
Age (years)	54(15)	59(12)	52(16)	<0.0001
Sex (male)	121(47.3)	32(37.6)	89(52)	0.0298
Body mass index (kg/m^2^)	24.4(3.84)	25.08(4.36)	23.81(3.64)	0.0029
AST (IU/L)	54.5(54.5)	87(82)	48(33)	<0.0001
ALT (IU/L)	73.5(82.5)	99(93)	64(69)	<0.0001
Total bilirubin (mg/dL)	0.91(0.41)	0.96(0.44)	0.89(0.43)	0.0466
Hemoglobin (g/dL)	13.9(2.05)	13.6(1.9)	14.1(2.2)	0.0812
γ-GT (IU/L)	37(51)	64(63)	30(35)	<0.0001
HCV genotype				0.5583
1, 4, 5, 6	163(63.7)	52(61.2)	111(64.9)	
2, 3	93(36.3)	33(38.8)	60(35.1)	
HCV RNA (log_10_ IU/mL)	6.32(1.22)	6.13(1.13)	6.36(1.22)	0.4875
IL-28B (rs8099917)				0.7810
T/G or G/G	34(13.3)	12(14.1)	22(12.9)	
T/T	222(86.7)	73(85.9)	149(87.1)	
IL-28B (rs12979860)				0.8864
C/T or T/T	38(14.8)	13(15.3)	25(14.6)	
C/C	218(85.2)	72(84.7)	146(85.4)	
LS (m/s)	1.48(0.89)	2.43(0.79)	1.26(0.32)	<0.0001
Decline in LS (m/s)	0.22(0.41)	0.66(0.69)	0.13(0.24)	<0.0001
METAVIR A grades				<0.0001
0	43(16.9)	1(1.2)	42(24.7)	
1	162(63.8)	47(56)	115(67.6)	
2	44(17.3)	32(38.1)	12(7.1)	
3	5(2)	4(4.8)	1(0.6)	
METAVIR F stages				<0.0001
1	93(36.6)	2(2.4)	91(53.5)	
2	110(43.3)	36(42.9)	74(43.5)	
3	29(11.4)	25(29.8)	4(2.4)	
4	22(8.7)	21(25)	1(0.6)	
Steatosis grades				0.4660
0, 1	247(96.5)	81(95.3)	166(97.1)	
2, 3	9(3.5)	4(4.7)	5(2.9)	
Platelet (×10^3^/μL)	168.5(81.5)	131(73)	181(76)	<0.0001
PT	1.02(0.11)	1.05(0.14)	1(0.1)	0.0003
APRI	0.81(1.31)	1.77(2.05)	0.68(0.72)	<0.0001
FIB-4	2.1(2.61)	3.83(3.61)	1.67(1.53)	<0.0001
Treatment (based)				0.1726
PegIFN for 24 weeks	108(42.2)	32(37.6)	76(44.4)	
PegIFN for 48 weeks	103(40.2)	41(48.2)	62(36.3)	
DAAs for 12 weeks	45(17.6)	12(14.1)	33(19.3)	
Ribavirin dose				0.1462
<80%	214(83.6)	67(78.8)	147(86)	
≥80%	42(16.4)	18(21.2)	24(14)	
RVR	156(60.9)	42(49.4)	114(66.7)	0.0077

LS, liver stiffness; AST, aspartate aminotransferase; ALT, alanine aminotransferase; γ-GT, γ-glutamyl transferase; IL-28B, interleukin-28B polymorphism; PT, prothrombin time (international normalized ratio); APRI, aspartate aminotransferase-to-platelet ratio index; pegIFN, pegylated interferon; DAA, direct-acting antiviral agent; RVR, rapid virologic response.

Of the 256 patients, 150 (60.9%) achieved RVR and 219 (85.5%) achieved SVR. Of the 45 patients who received DAA-based therapy, 44 (97.8%) achieved SVR.

The baseline values of age; sex; BMI; serum AST, ALT, and total bilirubin levels; γ-GT level; METAVIR A grades and F stages; platelet count; INR of PT; APRI and FIB-4; LS decline; and RVR differed significantly between the groups with baseline LSMs of <1.82 and ≥1.82 m/s (Table 1).

### Liver histology

Two patients did not undergo liver biopsies. Based on the METAVIR scoring system, 93 (36.6%), 110 (43.3%), 29 (11.4%), and 22 (8.7%) patients were staged as F1, F2, F3, and F4, respectively, and 43 (16.9%), 162 (63.8%), 44 (17.3%), and 5 (2%) patients were graded as A0, A1, A2, and A3, respectively, at baseline (Table 1). The Spearman correlation coefficients were 0.570 (*P* < 0.001) between baseline METAVIR F stages and FIB-4, 0.487 (*P* < 0.001) between F and APRI, and 0.619 (*P* < 0.001) between baseline METAVIR A grades and serum ALT.

### LSM

To dichotomize the METAVIR F stages by using LSMs, the optimal cut-off values for LS (m/s) were 1.62 for F1 versus F2–F4, 1.82 for F1 and F2 versus F3 and F4, and 1.86 for F1–F3 versus F4. Serum ALT levels were significantly (*P* < 0.0001) correlated with the LS estimated using univariate linear regression.

The paired LS values declined significantly from baseline to SVR visit in all groups and subgroups except the nonresponder subgroup (n = 10). Even in the group without SVR (n = 37) and the subgroup with relapse (n = 27), the paired LS values decreased significantly (Table 2). After stratification by milder versus advanced fibrosis stages and by lower and higher LS values, the LS decreased significantly. The LS declines were greater in the advanced fibrosis stages (METAVIR F3 and F4) than in the milder stages (F1 and F2) in either the SVR or non-SVR group (Table 2). Overall, approximately 80% of patients exhibited a decline in paired LS values in the stratified subgroups. The percentage of patients who exhibited decline did not differ significantly between the SVR (80.8%, 177/219), relapse (77.8%, 21/27), and nonresponder (80.0%, 8/10) groups (Table 2).

**Table 2:** Stratified changes in liver stiffness and serum alanine aminotransferase levels from baseline to sustained virologic response visit.

	Baseline		SVR visit		Changes from baseline to SVR visit			
	LS (m/s)	ALT (IU/L)	LS	ALT	Δ LS	Δ ALT	Δ LS (%)	Any decline in LS % (n/N)
Total (n = 256)	1.48(0.89)	73.5(82.5)	1.22(0.47)	20.0(15.0)	-0.22(0.41)*	-49.0(80.0)*	-15.0(22.6)	80.5(206/256)
METAVIR								
F1 (n = 93)	1.22(0.24)	57.0(53.0)	1.11(0.23)	17.0(10.0)	-0.11(0.22)*	-33.0(63.0)*	-9.2(17.0)	75.3(70/93)
F2 (n = 110)	1.56(0.71)	77.5(84.0)	1.23(0.41)	21.5(14.0)	-0.22(0.44)*	-52.5(82.0)*	-14.4(24.5)	81.8(90/110)
F3 (n = 29)	2.54(0.90)	145(130)	1.76(0.96)	26.0(28.0)	-0.50(0.69)*	-97.0(119)*	-25.4(18.5)	82.8(24/29)
F4 (n = 22)	2.62(1.04)	90.5(64.0)	1.96(0.90)	26.5(22.0)	-0.69(0.73)*	-55.5(53.0)*	-28.8(17.1)	90.9(20/22)
SVR								
Yes (n = 219)	1.43(0.76)	72.0(83.0)	1.21(0.42)	19.0(12.0)	-0.22(0.40)*	-53.0(85.0)*	-13.0(22.8)	80.8(177/219)
No (n = 37)	1.68(1.65)	76.0(67.0)	1.45(0.69)	47.0(43.0)	-0.17(0.43)*	-15.0(58.0)*	-15.0(22.6)	78.4(29/37)
Relapsers (n = 27)	1.68(1.73)	74.0(88.0)	1.45(0.69)	40.0(50.0)	-0.27(0.99)*	-25.0(67.0)*	-13.7(28.7)	77.8(21/27)
Non-responders (n = 10)	1.81(1.22)	79.5(40.0)	1.51(1.72)	61.0(49.0)	-0.13(0.35)	-9.0(13.0)*	-9.6(17.6)	80.0(8/10)
SVR (n = 219)								
F1, 2 (n = 176)	1.30(0.45)	67.0(78.0)	1.17(0.24)	17.5(10.0)	-0.16(0.34)*	-49.5(72.0)*	-12.4(20.9)	78.4(138/176)
F3, 4 (n = 41)	2.47(0.03)	99.0(101)	1.77(0.79)	25.0(15.0)	-0.59(0.56)*	-71.0(119)*	-26.3(18.5)	90.2(37/41)
LS <1.82 (n = 151)	1.25(0.31)	65.0(71.0)	1.14(0.19)	16.0(10.0)	-0.13(0.24)*	-48.0(67.5)*	-10.6(18.0)	77.5(117/151)
LS ≥1.82 (n = 68)	2.37(0.64)	99.5(108)	1.62(0.82)	24.5(12.5)	-0.68(0.60)*	-76.0(105)*	-30.3(24.9)	88.2(60/68)
Non SVR (n = 37)								
F1, 2 (n = 27)	1.54(0.78)	61.0(59.0)	1.39(0.54)	40.0(41.0)	-0.15(0.38)*	-12.0(41.0)*	-10.0(20.7)	81.5(22/27)
F3, 4 (n = 10)	3.32(0.61)	122(76.0)	2.17(1.46)	62.5(82.0)	-0.78(1.48)*	-36.5(59.0)*	-25.5(44.8)	70.0(7/10)
LS <1.82 (n = 20)	1.33(0.42)	60.5(40.0)	1.21(0.38)	38.5(25.5)	-0.10(0.22)*	-8.0(27.0)*	-8.1(14.9)	75(15/20)
LS ≥1.82 (n = 17)	3.01(0.92)	99.0(69.0)	1.94(1.15)	61.0(54.0)	-0.60(0.84)*	-24.0(52.0)*	-24.6(25.0)	82.4(14/17)

*P < 0.05; SVR, sustained virologic response; LS, liver stiffness; ALT, alanine aminotransferase.

**Fig 1:**
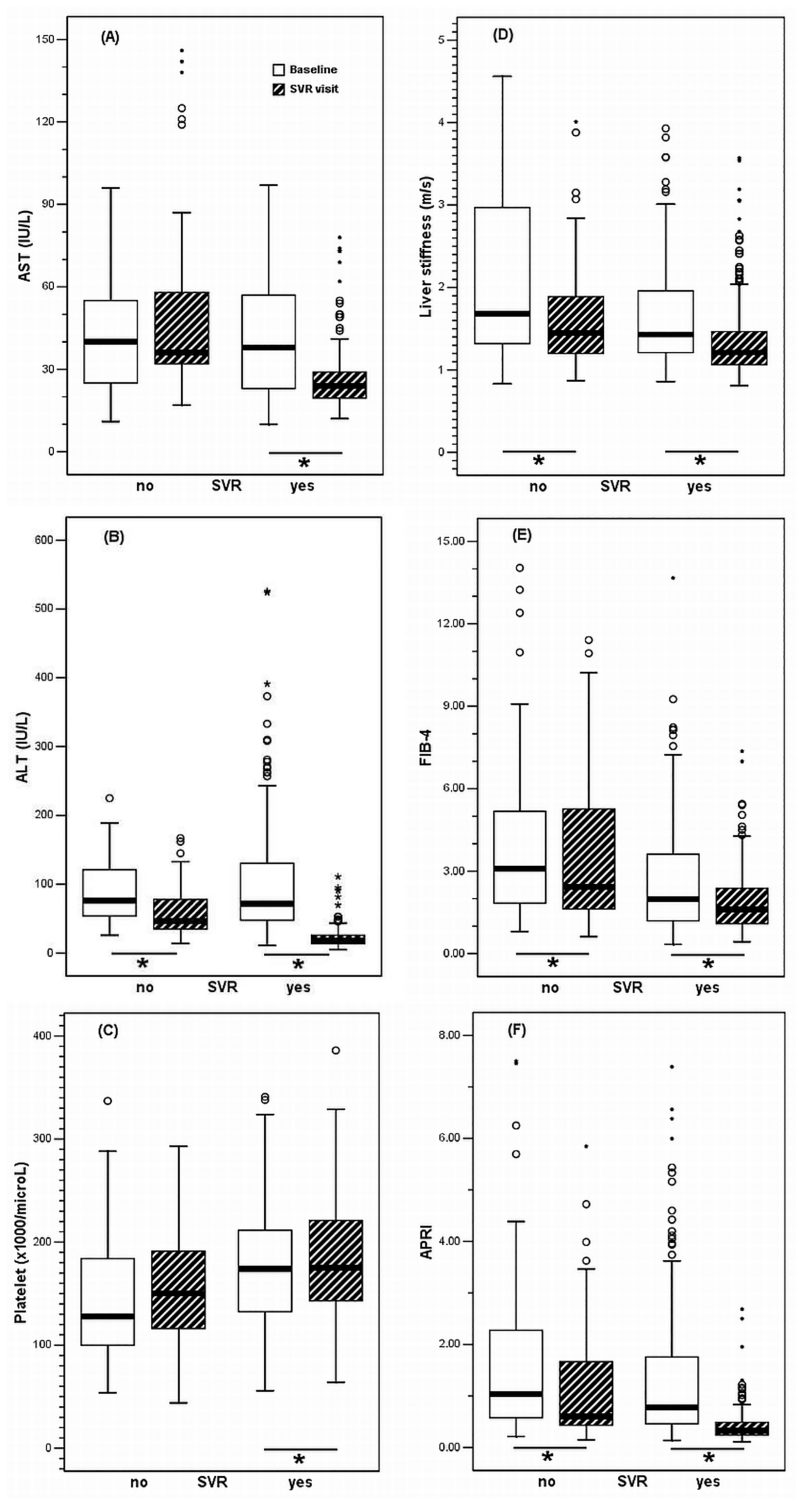
The paired values of aspartate aminotransferase (AST), alanine aminotransferase (ALT), platelet, liver stiffness, fibrosis-4 (FIB-4) index, and aspartate aminotransferase-to-platelet ratio index (APRI) compared between baseline and sustained virologic response (SVR) visit. SVR status: yes, n = 219; no, n = 37; *P < 0.05.

The results of ALT levels, the FIB-4 index, and APRI were consistent with those of the LSMs. The paired values of ALT, LS, the FIB-4 index, and APRI differed significantly from baseline to SVR visit (Fig 1; Tables 2 and 3).

**Table 3:** Stratified changes in FIB-4 index and aspartate aminotransferase-to-platelet ratio index from baseline to sustained virologic response visit.

	Baseline	SVR visit	Changes from baseline to SVR visit	*P* value
FIB-4				
Total (n = 256)	2.10(2.64)	1.68(1.43)	-0.29(1.26)	<0.0001
F1, 2 (n = 93)	1.77(1.65)	1.51(1.05)	-0.24(0.70)	<0.0001
F3, 4 (n = 110)	5.06(3.89)	2.91(2.15)	-1.37(2.77)	<0.0001
SVR (n = 219)	1.98(2.44)	1.62(1.28)	-0.30(1.24)	<0.0001
F1, 2 (n = 176)	1.69(1.53)	1.41(0.94)	-0.22(0.69)	<0.0001
F3, 4 (n = 41)	4.40(2.98)	2.87(1.36)	-1.42(2.22)	<0.0001
Non-SVR (n = 37)	3.10(3.34)	2.42(3.64)	-0.25(1.33)	<0.0001
F1, 2 (n = 27)	2.53(2.81)	2.19(1.19)	-0.25(1.02)	0.0113
F3, 4 (n = 10)	7.29(7.19)	8.97(7.99)	-0.58(5.84)	0.5566
Relapsers (n = 27)	3.10(3.09)	2.31(2.03)	-0.24(1.79)	0.1087
Non-responders (n = 10)	3.66(7.73)	3.18(6.61)	-0.27(0.63)	0.0840
APRI				
Total (n = 256)	0.81(1.31)	0.36(0.29)	-0.45(0.98)	<0.0001
F1, 2 (n = 93)	0.72(0.86)	0.31(0.19)	-0.38(0.72)	<0.0001
F3, 4 (n = 110)	2.13(2.68)	0.60(0.57)	-1.16(0.60)	<0.0001
SVR (n = 219)	0.78(1.31)	0.33(0.25)	-0.48(0.98)	<0.0001
F1, 2 (n = 176)	0.72(0.80)	0.30(0.15)	-0.41(0.75)	<0.0001
F3, 4 (n = 41)	2.13(1.98)	0.59(0.30)	-1.31(1.78)	<0.0001
Non-SVR (n = 37)	1.03(1.69)	0.60(1.23)	-0.21(1.00)	<0.0001
F1, 2 (n = 27)	0.82(1.00)	0.57(0.42)	-0.20(0.43)	0.0001
F3, 4 (n = 10)	2.56(4.08)	2.63(3.04)	-0.98(1.34)	0.2324
Relapsers (n = 27)	1.03(1.53)	0.58(0.61)	-0.21(1.11)	0.0048
Non-responders (n = 10)	1.15(2.45)	0.90(2.22)	-0.21(0.31)	0.0039

SVR, sustained virologic response; APRI, aspartate aminotransferase-to-platelet ratio index.

### Linear and logistic regressions for measurements

According to univariate and multiple linear regression analyses, BMI (*P* = 0.0062) and baseline LS (*P* < 0.0001) were identified as independent factors that explained LS decline (Table 4). For LS declines of > 4%, > 10%, and > 20% from the baseline, baseline LS remained independently significant (S1**–**S6 Tables). In the subgroup including only patients with baseline advanced fibrosis (METAVIR F3) or cirrhosis (F4), baseline LS remained independently significant (S7 and S8 Tables).

**Table 4:** Multiple linear regression analysis for declines in liver stiffness, FIB-4 and aspartate aminotransferase-to-platelet ratio index from baseline to sustained virologic response visit.

Covariates	Coefficient	Standard error of coefficient	*P* value
LS			
Body mass index (kg/m^2^)	-0.0181	0.0007	0.0062
Baseline LS (m/s)	0.3832	0.0301	<0.0001
FIB-4			
Baseline FIB-4	0.6452	0.0294	<0.0001
Baseline PT	-3.3245	0.9712	0.0007
SVR (yes versus no)	1.3516	0.2236	<0.0001
APRI			
Baseline APRI	0.7593	0.0230	<0.0001
Baseline PT	-0.6809	0.3985	0.0887
SVR (yes versus no)	0.7364	0.0894	<0.0001

LS, liver stiffness; PT, prothrombin time (international normalized ratio); SVR, sustained virologic response; APRI, aspartate aminotransferase-to-platelet ratio index.

In addition to baseline FIB-4 (*P* < 0.0001), baseline PT (*P* = 0.0007) and RVR (*P* < 0.0001) were identified as independent factors that explained the decline in the FIB-4 index (Table 4). Baseline APRI (*P* < 0.0001) and RVR (*P* < 0.0001) were identified as independent factors that explained the decline in APRI (Table 4).

### SVR correlates

Multiple binary logistic regression analysis identified interleukin-28B single nucleotide polymorphisms, baseline LS, and RVR as independent factors that correlated SVR to pegIFN-based therapy (Table 5).

**Table 5:** Multiple logistic regressions for sustained virologic response status.

	Total	Sustained virologic response		*P* value	Odds ratio	*P* value
		yes	no			
	(n = 211)	(n = 175)	(n = 36)			
Age (years)	54(15)	52(17)	60.5(8.5)	<0.0001		
Sex (male)	98(46.4)	85(48.6)	13(36.1)	0.1722		
Body mass index (kg/m^2^)	24.38(3.84)	24.36(4.06)	24.73(4.26)	0.2974	0.884(0.805–0.970)	0.0094
ALT (IU/L)	83(90)	85(91)	78.5(78)	0.5212		
Total bilirubin (mg/dL)	0.93(0.44)	0.93(0.44)	0.92(0.36)	0.8619		
Hemoglobin (g/dL)	13.8(2)	14(2)	13.1(1.85)	0.0169		
γ-glutamyl transferase (IU/L)	41(55)	40(52)	46(74)	0.1945		
HCV genotype				0.0054		
1, 4, 5, 6	120(56.9)	92(52.6)	28(77.8)			
2, 3	91(43.1)	83(47.4)	8(22.2)			
HCV RNA (log_10_ IU/mL)	6.23(1.23)	6.09(1.27)	6.49(1.12)	0.0107		
IL-28B (rs8099917)				0.0008		0.0058
T/G or G/G	28(13.3)	17(9.7)	11(30.6)		1.000	
T/T	183(86.7)	158(90.3)	25(69.4)		3.981(1.493–10.612)	
IL-28B (rs12979860)				0.0008		
C/T or T/T	32(15.2)	20(11.4)	12(33.3)			
C/C	179(84.8)	155(88.6)	24(66.7)			
Baseline LS (m/s)	1.52(0.97)	1.46(0.86)	1.74(1.69)	0.0327	1.000 (stiffness≥1.5)	
					4.223(2.178–8.187) (stiffness<1.5)	<0.0001
Decline in LS (m/s)	0.23(0.41)	0.23(0.4)	0.22(0.51)	0.9034		
METAVIR A grades				0.6307		
0, 1	162(77.5)	133(76.9)	29(80.6)			
2, 3	47(22.5)	40(23.1)	7(19.4)			
METAVIR F stages				0.1753		
1, 2	168(80.4)	142(82.1)	26(72.2)			
3, 4	41(19.6)	31(17.9)	10(27.8)			
Steatosis grades				0.6277		
0, 1	202(95.7)	167(95.4)	35(97.2)			
2, 3	9(4.3)	8(4.6)	1(2.8)			
Platelet (×10^3^/μL)	168(85)	175(84)	127(83)	0.0047		
PT	1.03(0.11)	1.03(0.11)	1.03(0.12)	0.5596		
APRI	0.9(1.44)	0.83(1.44)	1.09(1.87)	0.1620		
FIB-4	2.16(2.89)	2.09(2.53)	3.22(3.75)	0.0035		
Treatment (based)				<0.0001		
PegIFN for 24 weeks	108(51.2)	101(57.7)	7(19.4)			
PegIFN for 48 weeks	103(48.8)	74(42.3)	29(80.6)			
Ribavirin dose				0.0134		
<80%	176(83.4)	151(86.3)	25(69.4)			
≥80%	35(16.6)	24(13.7)	11(30.6)			
RVR (yes versus no)	113(53.6)	108(61.7)	5(13.9)	<0.0001	8.427(3.042–23.343)	<0.0001

ALT, alanine aminotransferase; IL-28B, interleukin-28B polymorphism; LS, liver stiffness; PT, prothrombin time (international normalized ratio); APRI, aspartate aminotransferase-to-platelet ratio index; pegIFN, pegylated interferon; RVR, rapid virologic response.

**Fig 2:**
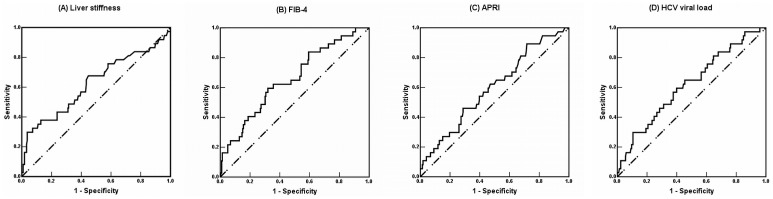
Receiver operating characteristic (ROC) curves using baseline liver stiffness (LS), fibrosis-4 (FIB-4) index, and the aspartate aminotransferase-to-platelet ratio index (APRI), and hepatitis C viral (HCV) load to dichotomize sustained virologic response (SVR) (no versus yes). According to the ROC analysis, the areas under curves were 0.624 (95% confidence interval, 0.514–0.733), 0.655 (0.560–0.751), 0.594 (0.496–0.692), and 0.618 (0.520–0.716) for baseline LS (A), the FIB-4 index (B), APRI (C), and HCV viral load (D), respectively, to dichotomize SVR (no versus yes). The comparison of the AUCs was not significant between the FIB-4 index and LS (*P* = 0.4231), between the APRI and LS (*P* = 0.4621), or between the HCV viral load and LS (*P* = 0.8104).

According to the ROC analysis, the AUCs were 0.624 (95% confidence interval, 0.514–0.733), 0.655 (0.560–0.751), 0.594 (0.496–0.692), and 0.618 (0.520–0.716) for baseline LS, the FIB-4 index, APRI, and HCV viral load, respectively, to dichotomize SVR (no versus yes). The comparison of the AUCs was not significant between the FIB-4 index and LS (*P* = 0.4231), between the APRI and LS (*P* = 0.4621), or between the HCV viral load and LS (*P* = 0.8104; Fig 2).

## Discussion

Despite the evolving modes of shear wave-based liver elastography, ARFI (point shear wave) elastography still possesses promising and comparable validity and reliability compared with other shear wave-based elastographies for evaluating bidirectional fibrogenesis models [13]. Moreover, the FIB-4 index and APRI are currently the two most widely used clinically accessible blood test-based indices for predicting liver fibrosis [19]. Our results support the role of the ARFI as a promising method for evaluating patients with CHC throughout antiviral therapy.

In the era of DAA-based treatments for CHC, patient cohorts receiving pegIFN with RBV are invaluable worldwide study resources for delineating the impact of various on- and off-treatment host and viral parameters on SVR. The liver pathology acquired at baseline of this study also provides invaluable parameters on liver tissue sections to correlate with post-SVR endpoints such as HCC occurrence in the long-term study [20].

Although most patients have shown growing reluctance toward paired liver biopsies over the years, previous studies or clinical trials using paired liver biopsies (pre- and post-antiviral treatment) have documented the rates of cirrhosis regression in 18%–64% of patients with cirrhosis over long-term intervals of up to 10 years [2–4]. In our study, of the 82 patients with baseline LS ≥ 1.86 m/s (the cut-off value for diagnosing cirrhosis in the present study), 43 (52.4%) exhibited regression (LS < 1.86 m/s at SVR visit) over significantly shorter periods than those reported in previous studies of patients with cirrhosis. However, follow-up liver biopsies were not performed in our cohort.

The present study and previous studies that have reported changes in LS over time can be compared in terms of the treatment course, decline, or kinetics of LS and other serum marker-based indices, as well as the factors associated with LS decline and correlates with SVR. In a previous study, of 328 patients who received DAA-based therapy and paired LSMs, 250 (76.2%) had exhibited improvement from baseline to SVR visit at 12 weeks after treatment [9]. In the present study, which employed baseline liver histology and paired LSMs, up to 80% of patients exhibited a decline in LS. Consistently, baseline fibrosis-relevant surrogates including the LS, FIB-4 and APRI independently and significantly explained the declines in these indices paired from baseline to the SVR visit (Table 4). Therefore, it is expected that these declines are greater in the advanced fibrosis stages than in the milder stages in either the SVR or non-SVR group. (Table 2), although the underlying mechanism remains unclear. Although the decline in LS in the present study was significantly correlated with and primarily explained by a decline in necroinflammation (Table 2) [8], the decline may be attributable to fibrosis reversal to some extent because of the follow-up intervals, which were longer than those reported in groups treated with DAA agents alone [9, 10]. In another study [5], significant declines in LSM values were reported in patient groups receiving pegIFN-based treatment who exhibited SVR and biochemical response (defined as monthly ALT levels under 30 IU/L lasting 24 months after the EOT).

One study reported that LS declined less significantly in the non-SVR group than in the SVR group [7]. This can be explained by the finding that aminotransferase levels did not significantly change in the non-SVR group over time [7]. Nonsignificant declines in paired ALT levels and LS values were also reported in the relapser group in one study that used DAA agents, where SVR was defined at 12 weeks after treatment [9]. By contrast, the paired LSM values in the present study declined significantly from baseline to SVR visit in all subgroups except the nonresponder subgroup (n = 10), in which paired LS values changed nonsignificantly from baseline to SVR (1.81 [1.22] to 1.51 [1.72] m/s), although the paired ALT levels exhibited a significant decline (79.5 [40.0] to 61.0 [49.0] IU/L; Table 2). These LS decline patterns in the present study are consistent with and indirectly verified by those measured using the FIB-4 index and APRI in the same cohort (Table 3) and can be best explained by the observation that the relapser group had normalized ALT during post-treatment follow-up despite failing therapy.

Throughout multiple regressions, baseline LSM values outweighed other factors, including the baseline HCV viral load and serum ALT levels, to predict LS decline (Table 4). These results are consistent with those reported in previous studies [5–7, 9, 10]. LS decline did not significantly correlate with SVR (Table 5), as explained by the significant LS decline in both the SVR and non-SVR groups (Table 2). However, treatment responses explained the declines in the FIB-4 index and APRI in the present study (Table 4). These findings still cannot be reconciled by the data acquired in the present study or explained by previous reports that platelet counts are affected by necroinflammatory degrees, the use of erythropoietin, inosine triphosphate pyrophosphatase polymorphisms, and anemia [21].

Despite the finding that the declines of serum ALT levels correlated significantly with those of LS over time (Table 2), univariate or multiple linear regressions for any LS decline did not identify baseline ALT levels or ALT decline as significant correlates (Table 4). However, the effect of necroinflammation on LS decline can still be observed, with baseline ALT levels significantly correlating with LS declines of > 10% and > 20% in the univariate analyses (S3 and S5 Tables). However, the effects did not retain significance after the final multiple regressions as shown in another study [22].

This study has some limitations. First, because of the lack of paired or serial liver histology, delineating pathological changes or reversals in the fibrosis stages or activity grades during on- and off-treatment periods is infeasible [23]. Future correlation studies with liver biopsies are warranted to elucidate the measured LS declines that can be attributed to the resolution of necroinflammation or fibrosis reversal. Second, for the heterogeneous durations, the brief treatment course in the DAA-based group actually reflected necroinflammatory declines approximate to those in the pegIFN-based group. Therefore, combining these two groups to delineate the reported declines is a valid practice. Continual future surveillance of our present cohort is warranted to elucidate the long-term changes in the LS. Third, the present study lacks comparison with a large patient group exhibiting natural limited improvements or even exacerbations over an untreated period. In addition, in this study, patients with serial LSMs who discontinued treatment were not recruited. Fourth, the present study delineated chronological changes and evaluated the significance of associations instead of conducting time-dependent statistics as bases for future surveillance of the present cohort. Fifth, LSMs at the EOT were lacking in the present study. However, the time interval between the EOT and SVR visit was significantly shorter than that reported in studies with follow-up periods of up to 10 years. In terms of the process of fibrosis reversal, the EOT status may not significantly differ from that at SVR visit [10].

In conclusion, paired LSMs in patient groups treated for CHC revealed favorable results consistent with those of the FIB-4 index and APRI. Most patients benefited from the treatment and exhibited significant declines in all three diagnostic measurements or indices. The declines may have correlated with necroinflammatory declines. SVR was predicted by the baseline rather than the declines of the three diagnostic measurements.

## Supporting information

S1 Table Univariate analysis for decline >4% in liver stiffness from baseline to sustained virologic response visit. (DOC)

S2 Table Multiple logistic regression for liver stiffness decline >4%. (DOC)

S3 Table Univariate analysis for decline >10% in liver stiffness from baseline to sustained virologic response visit. (DOC)

S4 Table Multiple logistic regression for LS decline >10%. (DOC)

S5 Table Univariate analysis for decline >20% in liver stiffness from baseline to sustained virologic response visit. (DOC)

S6 Table Multiple logistic regression for liver stiffness decline >20%. (DOC)

S7 Table Univariate linear regression for liver stiffness decline in patients with advanced fibrosis and cirrhosis (n = 51). (DOC)

S8 Table Multiple linear regression for liver stiffness decline in patients with advanced fibrosis and cirrhosis (n = 51). (DOC)
