# Supplementary material for: Changes in liver stiffness measurement using acoustic radiation force impulse elastography after antiviral therapy in patients with chronic hepatitis C
Source: PLoS One. 2018 Jan 2;13(1):e0190455. doi: 10.1371/journal.pone.0190455 (PMC5749809; doi:10.1371/journal.pone.0190455)
Supplement: S1 Table — (DOC) [file pone.0190455.s001.doc]

**S1 Table. Univariate analysis for decline** >4% **in liver stiffness from baseline to sustained virologic response visit.**

| Variables | Total | Yes | No |  |
| --- | --- | --- | --- | --- |
| Median (IQR) or n (%) | (n=256) | (n=192) | (n=64) | *P* value |
| Age (years) | 54(15) | 55(16) | 53(14.5) | 0.6657 |
| Sex (male) | 121(47.3) | 87(45.3) | 34(53.1) | 0.2783 |
| Body mass index (kg/m^2^) | 24.4(3.84) | 24.36(3.95) | 24.66(4.46) | 0.3837 |
| ALT (IU/L) | 73.5(82.5) | 75.5(86.5) | 65.5(57) | 0.1527 |
| Total bilirubin (mg/dL) | 0.91(0.41) | 0.91(0.43) | 0.9(0.4) | 0.3787 |
| Hemoglobin (g/dL) | 13.85(2.05) | 13.75(2) | 14.2(2) | 0.1149 |
| γ-GT (IU/L) | 37(51) | 41(50) | 32.5(59) | 0.3108 |
| Genotype |  |  |  | 0.1151 |
| 1, 4, 5, 6 | 163(63.7) | 117(60.9) | 46(71.9) |  |
| 2, 3 | 93(36.3) | 75(39.1) | 18(28.1) |  |
| HCV RNA (log_10_IU/mL) | 6.32(1.22) | 6.33(1.23) | 6.25(1.21) | 0.8517 |
| IL-28B (rs8099917) |  |  |  | 0.0556 |
| T/G or G/G | 34(13.3) | 21(10.9) | 13(20.3) |  |
| T/T | 222(86.7) | 171(89.1) | 51(79.7) |  |
| IL-28B (rs12979860) |  |  |  | 0.0256 |
| C/T or T/T | 38(14.8) | 23(12) | 15(23.4) |  |
| C/C | 218(85.2) | 169(88) | 49(76.6) |  |
| LS (m/s) | 1.48(0.89) | 1.57(0.92) | 1.24(0.41) | <0.0001 |
| METAVIR A grades |  |  |  | 0.1114 |
| 0, 1 | 205(80.7) | 149(78.4) | 56(87.5) |  |
| 2, 3 | 49(19.3) | 41(21.6) | 8(12.5) |  |
| METAVIR F stages |  |  |  | 0.0801 |
| 1, 2 | 203(79.9) | 147(77.4) | 56(87.5) |  |
| 3, 4 | 51(20.1) | 43(22.6) | 8(12.5) |  |
| Steatosis grades |  |  |  | 0.5567 |
| 0, 1 | 247(96.5) | 186(96.9) | 61(95.3) |  |
| 2, 3 | 9(3.5) | 6(3.1) | 3(4.7) |  |
| Platelet (×10^3^/μL) | 168.5(81.5) | 162(84) | 177(79.5) | 0.1247 |
| PT | 1.02(0.11) | 1.03(0.1) | 1(0.08) | 0.0004 |
| APRI | 0.81(1.31) | 0.88(1.37) | 0.72(1.11) | 0.0670 |
| FIB-4 | 2.1(2.61) | 2.16(2.79) | 1.8(2) | 0.1252 |
| Treatment (based) |  |  |  | 0.1414 |
| PegIFN for 24 weeks | 108(42.2) | 86(44.8) | 22(34.4) |  |
| PegIFN for 48 weeks | 103(40.2) | 77(40.1) | 26(40.6) |  |
| DAAs for 12 weeks | 45(17.6) | 29(15.1) | 16(25) |  |
| Ribavirin dose |  |  |  | 0.8455 |
| <80% | 214(83.6) | 160(83.3) | 54(84.4) |  |
| ≥80% | 42(16.4) | 32(16.7) | 10(15.6) |  |

LS, liver stiffness; ALT, alanine aminotransferase; γ-GT, γ-glutamyl transferase; IL-28B, interleukin-28B polymorphism; PT, prothrombin time (international normalized ratio); APRI, aspartate aminotransferase-to-platelet ratio index; peg-IFN, pegylated interferon; DAA, direct-acting antiviral agent
