# Supplementary material for: Changes in liver stiffness measurement using acoustic radiation force impulse elastography after antiviral therapy in patients with chronic hepatitis C
Source: PLoS One. 2018 Jan 2;13(1):e0190455. doi: 10.1371/journal.pone.0190455 (PMC5749809; doi:10.1371/journal.pone.0190455)
Supplement: S3 Table — (DOC) [file pone.0190455.s003.doc]

**S3 Table. Univariate analysis for decline >10% in liver stiffness from baseline to sustained virologic response visit.**

| Variables | Total | Yes | No |  |
| --- | --- | --- | --- | --- |
| Median (IQR) or n (%) | (n=256) | (n=156) | (n=100) | *P* value |
| Age (years) | 54(15) | 55.5(15) | 52(17) | 0.1942 |
| Sex (male) | 121(47.3) | 67(42.9) | 54(54) | 0.0840 |
| Body mass index (kg/m^2^) | 24.4(3.84) | 24.46(3.75) | 24.07(3.78) | 0.4488 |
| ALT (IU/L) | 73.5(82.5) | 78.5(85.5) | 66(76) | 0.0436 |
| Total bilirubin (mg/dL) | 0.91(0.41) | 0.92(0.44) | 0.9(0.48) | 0.0666 |
| Hemoglobin (g/dL) | 13.85(2.05) | 13.7(1.85) | 14.2(2.35) | 0.1195 |
| γ-GT (IU/L) | 37(51) | 45(50) | 32.5(52) | 0.0425 |
| Genotype |  |  |  | 0.0919 |
| 1, 4, 5, 6 | 163(63.7) | 93(59.6) | 70(70) |  |
| 2, 3 | 93(36.3) | 63(40.4) | 30(30) |  |
| HCV RNA (log_10_IU/mL) | 6.32(1.22) | 6.28(1.22) | 6.35(1.21) | 0.8002 |
| IL-28B (rs8099917) |  |  |  | 0.1604 |
| T/G or G/G | 34(13.3) | 17(10.9) | 17(17) |  |
| T/T | 222(86.7) | 139(89.1) | 83(83) |  |
| IL-28B (rs12979860) |  |  |  | 0.1343 |
| C/T or T/T | 38(14.8) | 19(12.2) | 19(19) |  |
| C/C | 218(85.2) | 137(87.8) | 81(81) |  |
| LS (m/s) | 1.48(0.89) | 1.69(0.91) | 1.24(0.33) | <0.0001 |
| METAVIR A grades |  |  |  | 0.0406 |
| 0, 1 | 205(80.7) | 118(76.6) | 87(87) |  |
| 2, 3 | 49(19.3) | 36(23.4) | 13(13) |  |
| METAVIR F stages |  |  |  | 0.0012 |
| 1, 2 | 203(79.9) | 113(73.4) | 90(90) |  |
| 3, 4 | 51(20.1) | 41(26.6) | 10(10) |  |
| Steatosis grades |  |  |  | 0.7362 |
| 0, 1 | 247(96.5) | 151(96.8) | 96(96) |  |
| 2, 3 | 9(3.5) | 5(3.2) | 4(4) |  |
| Platelet (×10^3^/μL) | 168.5(81.5) | 155.5(76.5) | 180(81.5) | 0.0024 |
| PT | 1.02(0.11) | 1.03(0.1) | 1(0.09) | 0.0014 |
| APRI | 0.81(1.31) | 0.96(1.35) | 0.66(1.02) | 0.0010 |
| FIB-4 | 2.1(2.61) | 2.32(2.68) | 1.62(2.14) | 0.0013 |
| Treatment (based) |  |  |  | 0.2350 |
| PegIFN for 24 weeks | 108(42.2) | 71(45.5) | 37(37) |  |
| PegIFN for 48 weeks | 103(40.2) | 62(39.7) | 41(41) |  |
| DAAs for 12 weeks | 45(17.6) | 23(14.7) | 22(22) |  |
| Ribavirin dose |  |  |  | 0.8373 |
| <80% | 214(83.6) | 131(84) | 83(83) |  |
| ≥80% | 42(16.4) | 25(16) | 17(17) |  |

LS, liver stiffness; ALT, alanine aminotransferase; γ-GT, γ-glutamyl transferase; IL-28B, interleukin-28B polymorphism; PT, prothrombin time (international normalized ratio); APRI, aspartate aminotransferase-to-platelet ratio index; peg-IFN, pegylated interferon; DAA, direct-acting antiviral agent
