# Supplementary material for: Changes in liver stiffness measurement using acoustic radiation force impulse elastography after antiviral therapy in patients with chronic hepatitis C
Source: PLoS One. 2018 Jan 2;13(1):e0190455. doi: 10.1371/journal.pone.0190455 (PMC5749809; doi:10.1371/journal.pone.0190455)
Supplement: S4 Table — (DOC) [file pone.0190455.s004.doc]

**S4 Table. Multiple logistic regression for LS decline >10%**.

|  | Odds ratio (95% Confidence interval) | *P* value |
| --- | --- | --- |
| Sex |  | 0.1503 |
| Female | 1.497(0.864-2.594) |  |
| Male | 1.000 |  |
| Liver stiffness (m/s) |  | <0.0001 |
| <1.5 | 1.000 |  |
| ≥1.5 | 5.765(3.267-10.144) |  |
