# Supplementary material for: Changes in liver stiffness measurement using acoustic radiation force impulse elastography after antiviral therapy in patients with chronic hepatitis C
Source: PLoS One. 2018 Jan 2;13(1):e0190455. doi: 10.1371/journal.pone.0190455 (PMC5749809; doi:10.1371/journal.pone.0190455)
Supplement: S5 Table — (DOC) [file pone.0190455.s005.doc]

**S5 Table. Univariate analysis for decline >20% in liver stiffness from baseline to sustained virologic response visit.**

| Variables | Total | Yes | No |  |
| --- | --- | --- | --- | --- |
| Median (IQR) or n (%) | (n=256) | (n=94) | (n=162) | *P* value |
| Age (years) | 54(15) | 56(14) | 53.5(17) | 0.1528 |
| Sex (male) | 121(47.3) | 37(39.4) | 84(51.9) | 0.0537 |
| Body mass index (kg/m^2^) | 24.4(3.84) | 24.47(3.61) | 24.11(4.12) | 0.3280 |
| ALT (IU/L) | 73.5(82.5) | 86(95) | 65.5(78) | 0.0045 |
| Total bilirubin (mg/dL) | 0.91(0.41) | 0.92(0.39) | 0.9(0.42) | 0.8990 |
| Hemoglobin (g/dL) | 13.85(2.05) | 13.75(1.7) | 14(2.4) | 0.7948 |
| γ-GT (IU/L) | 37(51) | 52(57) | 32(39) | 0.0004 |
| Genotype |  |  |  | 0.2991 |
| 1, 4, 5, 6 | 163(63.7) | 56(59.6) | 107(66) |  |
| 2, 3 | 93(36.3) | 38(40.4) | 55(34) |  |
| HCV RNA (log_10_IU/mL) | 6.32(1.22) | 6.28(1.08) | 6.33(1.3) | 0.5295 |
| IL-28B (rs8099917) |  |  |  | 0.1831 |
| T/G or G/G | 34(13.3) | 9(9.6) | 25(15.4) |  |
| T/T | 222(86.7) | 85(90.4) | 137(84.6) |  |
| IL-28B (rs12979860) |  |  |  | 0.1494 |
| C/T or T/T | 38(14.8) | 10(10.6) | 28(17.3) |  |
| C/C | 218(85.2) | 84(89.4) | 134(82.7) |  |
| LS (m/s) | 1.48(0.89) | 1.98(1.01) | 1.27(0.38) | <00001 |
| METAVIR A grades |  |  |  | 0.0003 |
| 0-1 | 205(80.7) | 64(68.8) | 141(87.6) |  |
| 2-3 | 49(19.3) | 29(31.2) | 20(12.4) |  |
| METAVIR F stages |  |  |  | <00001 |
| 0-2 | 203(79.9) | 61(65.6) | 142(88.2) |  |
| 3-4 | 51(20.1) | 32(34.4) | 19(11.8) |  |
| Steatosis grades |  |  |  | 0.3584 |
| 0-1 | 247(96.5) | 92(97.9) | 155(95.7) | . |
| 2-3 | 9(3.5) | 2(2.1) | 7(4.3) | . |
| Platelet (×10^3^/μL) | 168.5(81.5) | 144(67) | 177.5(80) | 0.0006 |
| PT | 1.02(0.11) | 1.03(0.1) | 1.01(0.11) | 0.0883 |
| APRI | 0.81(1.31) | 1.25(1.54) | 0.71(1) | 0.0000 |
| FIB-4 | 2.1(2.61) | 2.85(2.56) | 1.72(1.94) | 0.0001 |
| Treatment (based) |  |  |  | 0.4461 |
| PegIFN for 24 weeks | 108(42.2) | 40(42.6) | 68(42) |  |
| PegIFN for 48 weeks | 103(40.2) | 41(43.6) | 62(38.3) |  |
| DAAs for 12 weeks | 45(17.6) | 13(13.8) | 32(19.8) |  |
| Ribavirin dose |  |  |  | 0.3965 |
| <80% | 214(83.6) | 81(86.2) | 133(82.1) |  |
| ≥80% | 42(16.4) | 13(13.8) | 29(17.9) |  |

LS, liver stiffness; ALT, alanine aminotransferase; γ-GT, γ-glutamyl transferase; IL-28B, interleukin-28B polymorphism; PT, prothrombin time (international normalized ratio); APRI, aspartate aminotransferase-to-platelet ratio index; peg-IFN, pegylated interferon; DAA, direct-acting antiviral agent
