# Supplementary material for: Changes in liver stiffness measurement using acoustic radiation force impulse elastography after antiviral therapy in patients with chronic hepatitis C
Source: PLoS One. 2018 Jan 2;13(1):e0190455. doi: 10.1371/journal.pone.0190455 (PMC5749809; doi:10.1371/journal.pone.0190455)
Supplement: S7 Table — (DOC) [file pone.0190455.s007.doc]

**S7 Table. Univariate linear regression for liver stiffness decline in patients with advanced fibrosis and cirrhosis (n=51)**.

| Variables | Coefficient | SE | *P* value |
| --- | --- | --- | --- |
| Age (years) | -0.0068 | 0.0085 | 0.4277 |
| Sex (male versus female) | 0.0129 | 0.1605 | 0.9363 |
| Body mass index (kg/m^2^) | -0.0144 | 0.0214 | 0.5047 |
| AST (IU/L) | -0.0016 | 0.0013 | 0.2525 |
| AST decline (IU/L) | -0.0012 | 0.0014 | 0.3759 |
| ALT (IU/L) | -0.0011 | 0.0011 | 0.3201 |
| ALT decline (IU/L) | -0.0008 | 0.0011 | 0.4614 |
| Total bilirubin (mg/dL) | -0.1340 | 0.2212 | 0.5476 |
| Total bilirubin decline (mg/dL) | 0.0892 | 0.1614 | 0.5831 |
| Hemoglobin (g/dL) | 0.0177 | 0.0586 | 0.7641 |
| γ-GT | -0.0012 | 0.0011 | 0.2556 |
| HCV genotypes: 1,4,5,6 versus 2,3 | -0.0823 | 0.1567 | 0.6017 |
| HCV RNA (log_10_ copies/mL) | -0.0410 | 0.0804 | 0.6120 |
| HCV RNA decline (log_10_ copies/mL) | -0.0640 | 0.0778 | 0.4143 |
| IL-28B (rs8099917): T/T versus non T/T | 0.1823 | 0.2842 | 0.5241 |
| IL-28B (rs12979860): C/C versus non C/C | 0.3116 | 0.2541 | 0.2260 |
| LS (m/s) | 0.2644 | 0.0991 | 0.0103 |
| METAVIR A grades: 2,3 versus 0,1 | -0.0389 | 0.1534 | 0.8008 |
| Platelet (×10^3^/μL) | -0.0007 | 0.0015 | 0.6116 |
| Platelet increase (×10^3^/μL) | -0.0002 | 0.0016 | 0.9016 |
| PT | 0.3072 | 0.7645 | 0.6896 |
| PT decline | 1.7543 | 0.9389 | 0.0677 |
| Treatment: Peg-INF versus DAA | 0.0292 | 0.1932 | 0.8806 |
| SVR: yes versus no | -0.0945 | 0.1928 | 0.6262 |

SE, standard error of coefficient; LS, liver stiffness; AST, aspartate aminotransferase; ALT, alanine aminotransferase; γ-GT, γ-glutamyl transferase; IL-28B, interleukin-28B polymorphism; PT, prothrombin time (international normalized ratio); APRI, aspartate aminotransferase-to-platelet ratio index; peg-IFN, pegylated interferon; DAA, direct-acting antiviral agent; SVR, sustained virologic response
