# Supplementary material for: Changes in liver stiffness measurement using acoustic radiation force impulse elastography after antiviral therapy in patients with chronic hepatitis C
Source: PLoS One. 2018 Jan 2;13(1):e0190455. doi: 10.1371/journal.pone.0190455 (PMC5749809; doi:10.1371/journal.pone.0190455)
Supplement: S8 Table — (DOC) [file pone.0190455.s008.doc]

**S8 Table. Multiple linear regression for liver stiffness decline** **in patients with advanced fibrosis and cirrhosis (n=51).**

| Variables | Coefficient | SE | *P* value |
| --- | --- | --- | --- |
| Body mass index (kg/m^2^) | -0.0330 | 0.0207 | 0.1169 |
| Liver stiffness (m/s) | 0.3125 | 0.1021 | 0.0036 |

SE, standard error of coefficient
